# Potential reproductive health effects and oxidative stress associated with exposure to potassium dichromate (K_2_Cr_2_O_7_) and magnesium sulphate (MgSO_4_) in male mice

**DOI:** 10.12669/pjms.304.4757

**Published:** 2014

**Authors:** Mahmood Rasool, Kalsoom Zaigham, Arif Malik, Muhammad Imran Naseer, Umm-e-Habiba, Abdul Manan, Mahmood Husain Qazi, Muhammad Asif

**Affiliations:** 1 Mahmood Rasool, Center of Excellence in Genomic Medicine Research (CEGMR), King Abdulaziz University, Jeddah, Saudi Arabia.; 2 Kalsoom Zaigham, Center of Excellence in Genomic Medicine Research (CEGMR), King Abdulaziz University, Jeddah, Saudi Arabia.; 3 Arif Malik, Center of Excellence in Genomic Medicine Research (CEGMR), King Abdulaziz University, Jeddah, Saudi Arabia.; 4 Muhammad Imran Naseer, Center of Excellence in Genomic Medicine Research (CEGMR), King Abdulaziz University, Jeddah, Saudi Arabia.; 5 Umm-e-Habiba, Institute of Molecular Biology and Biotechnology, The University of Lahore, Lahore, Pakistan.; 6 Abdul Manan, Institute of Molecular Biology and Biotechnology, The University of Lahore, Lahore, Pakistan.; 7 Mahmood Husain Qazi, Centre for Research in Molecular Medicine, The University of Lahore, Pakistan.; 8 Muhammad Asif, Department of Biotechnology and Informatics, BUITEMS, Quetta, Pakistan.

**Keywords:** Magnesium sulphate (MgSO4), Malondialdehyde (MDA), Oxidative stress, Potassium dichromate (K2Cr2O7), Superoxide dismutase (SOD)

## Abstract

***Objective:*** To investigate the potential harmful effects of potassium dichromate and magnesium sulphate causing oxidative stress and reproductive toxicity in adult male mice model.

***Methods:*** The experimental work was conducted on sixty male mice (*Mus musculus*) divided into three groups. Mice in group B and C received potassium dichromate and magnesium sulphate of 5.0 and 500 mg/Kg body weight/ml respectively, for sixty days. The blood sample was analyzed to assess oxidative stress and cellular damage.

***Results:*** Results showed high malondialdehyde (MDA) and low levels of antioxidant enzymes [catalase (CAT), superoxide dismutase (SOD) and glutathione peroxidase (GPx)] in both potassium dichromate and magnesium sulphate administrated groups as compared to control group. Reduced number of sperm count and excessive destruction of testicular follicles, including destruction of spermatids, leydig cells and sertoli cells, were also seen in both groups.

***Conclusion:*** We concluded from present study that potassium dichromate and magnesium sulphate causes oxidative stress by generation of reactive oxygen species (ROS) and causing DNA damage in testicular cells leading to adverse reproductive abnormalities.

## INTRODUCTION

Worldwide distribution and extensive use of chemical agents, is associated with concern of the highest priority for environmental and industrial exposure which may have imposing effects on male reproductive function. Chromium has been identified to be one of these toxic metals. Its common salt, potassium dichromate (K2Cr2O7) is most commonly used as an oxidizing agent in various laboratory and industrial applications.^1^^,^^2^ Chromium VI is more toxic than in trivalent form because it readily enters the cells producing various pathological conditions, including reproductive dysfunction, induce toxicity provoke lipid peroxidation, DNA damage, cytotoxicity, mutagenesis and carcinogenesis.^3^ Magnesium sulfate (MgSO4) is a chemical compound often encountered as the heptahydrate epsomite (MgSO4·7H_2_O) commonly called Epsom salt, is used as a first line of treatment in the majority of cases. It is used in replacement therapy for hypomagnesaemia and as a bronchodilator in severe exacerbations of asthma. It is commonly administered via the intravenous route for the management of severe asthma attacks.^4^^,^^5^

Lipid peroxidation is one of the major outcomes of free radical-mediated to cellular injury or beneficial biological effects. K2Cr2O7 and MgSO4 both induce oxidative stress due to which high lipid peroxidation occurs and in result MDA level is increased and antioxidant enzymes SOD and CAT activity is decreased.^6^^,^^7^ The evidence indicates that the careless use of toxic heavy metals in the past twenty years have shown very alarming trend in male reproductive health.^8^ Testicular tissues are major target organ for metals that induce oxidative damage because of its high contents of polyunsaturated membrane lipids. Ingestion of hexavalent chromium compounds produces uncertain levels of degeneration in the outer most cellular layers in several somniferous tubules, reducing the number of sperm count and spermatogonia per tubule, leading to considerable increases in the morphologically abnormal sperms percentage.^9^^,^^10^ Oral administration of vanadyl sulphate for 60 days caused a decrease in the weights of testes, accessory reproductive organs and the diameter of seminiferous tubules and leydig cells nuclei are reduced.^11^ The aim of this research work was to check the adverse effects of K2Cr2O7 and MgSO4 on the testes of adult male mice.

## METHODS

***Experimental design: ***Sixty adult male mice were included in the study, divided into three groups, comprising twenty mice in each group with average weight ranging from 25-40 gm. Group A served as control, while in group B mice were treated with K_2_Cr_2_O_7 _and in group C mice were administered with MgSO_4_. All the laboratory work was performed at the Institute of Molecular Biology & Biotechnology, the University of Lahore, during March 2013 to September 2013.

***Administration of K***_2_ ***Cr***_2_ ***O***_7_ ***and MgSO***_4_ ***: ***K_2_Cr_2_O_7 _was administrated at the dose of 5mg/Kg body weight while MgSO_4 _was administrated at the dose of 500mg/Kg body weight.^12^ Both the salts were administrated orally for the period of 60 days.

***Samples collection and sample analysis: ***2 ml blood was taken from each mouse at 1^st^, 30^th^ and 60^th^ day of the experiment and serum of the sample was separated by centrifugation at 3000 rpm. Then sample were processed and analyzed for the estimation of MDA, SOD, CAT and GPx activity.

***Estimation of*** ***MDA*** ***: ***Thiobarbituric acid reactive substance test was used for the estimation of MDA level in serum.^13^ Total 1ml of serum was taken and a 10% (w/v) homogenate was prepared in 10 mM buffer (pH 7.4). The supernatant was used for immediate thiobarbituric acid reactive substances test. In this test 200µl of serum sample, 200µl of 8.1% sodium dodecyl sulfate (SDS), 1.5ml of 0.8% TBA, 1.5ml of 20% acetic acid solution (pH 3.5) and 4.0ml distilled water and 5.0 ml of n-butanol were used and absorbance at 532nm was recorded.

***Estimation of SOD: ***All procedure for estimation of SOD was performed in ice bath.^14^ Blood serum was taken and homogenate was prepared in 50% TCA and centrifuged at 13000 rpm. The supernatant was used for immediate SOD and other enzyme activity evaluation. 100µl of serum sample, 1.2ml of sodium phosphate buffer (pH 8.3, 0.052M), 100µl of phenazine methosulphate (186µm), 300 µl of nitro blue tetrazolium (300µm), 200 µl of NADH (750µm) and 4.0 ml of n- butanol were used and absorbance was recorded at 560nm.

***Estimation of CAT activity: ***Catalase activity was measured by the method of Aebi.^15^ The supernatant was used for the estimation of catalase. The rate of decomposition of H2O2 was measured at 240nm.

***Estimation of GPx activity: ***Glutathione peroxidase was determined by homogenizing 0.1 ml of serum with 2.4ml of 0.02 M EDTA first and then the test tubes were kept in an ice bath for 10 minutes. 2ml of distilled water and 0.5 ml of TCA (50%) was added and was kept in an ice bath for 10-15 minutes. The mixture was centrifuged at 3000-3500rpm for 10 minutes. 1ml of the supernatant was taken in test tube and 2ml of 0.15M Tris HCL plus 0.05ml DTNB were added in it and absorbance was taken at 412nm.

***Histopathology of testicles:*** Two mice from each group were randomly selected for histopathology of testis at 1^st^, 30^th^ and 60^th^ day of the experiment. These mice were sacrificed and the specimens from testicular tissues were fixed in 10% neutral buffer formalin, dehydrated in ascending grades of ethanol alcohols, cleared in xylol, casted, blocked, cut at 2-5 μm thickness and stained with hematoxylin-eosin for microscopic examination.^16^

***Statistical analysis: ***All the data thus obtained was statistically analyzed by applying one way analysis of variance (ANOVA). The differences of the means were considered significant at p < 0.05.

## RESULTS

***MDA levels in male mice receiving K***_2_ ***Cr***_2_ ***O***_7_ ***and MgSO***_4_ ***: ***MDA levels in different groups at different days were studied. It was concluded from the descriptive statistics that at first day insignificant differences in MDA levels between all the groups were observed as compared to control group (Table-Ia). While significant differences in MDA levels were observed in all the groups at 30^th^ and 60^th^ day as compared to control group. The results showed that both of these salts cause significant elevation in MDA levels, resulting in high lipid peroxidation.

***SOD levels in male mice receiving K***_2_ ***Cr***_2_ ***O***_7_ ***and MgSO***_4_ ***: ***Further, SOD levels in different groups at different days were studied. The SOD level decreased significantly in the groups B and C at 30^th^ and 60^th^ day as compared to control groups (Table-Ib). It is concluded that both of these salts cause significant depletion of SOD levels, resulting in high lipid peroxidation. SOD level in MgSO_4_ treated mice is even lower than with K_2_Cr_2_O_7 _treated mice.

***Levels of CAT in male mice receiving K***_2_ ***Cr***_2_ ***O***_7_ ***and MgSO***_4_ ***:*** Table-IIa shows the CAT levels in different groups at different days. Significant differences in CAT levels were observed in all the groups at 60^th^ day as compared to control group. CAT level was found to be more reduced with MgSO_4 _as compared to K_2_Cr_2_O_7_.

***GP*** ***x*** ***level in male mice receiving K***_2_ ***Cr***_2_ ***O***_7_ ***and MgSO***_4_ ***:*** Table-IIb shows the GPx levels in different groups at different days. Significant differences in GPx levels were observed in all the groups at 30^th^ and 60^th^ day as compared to control group. So it was concluded that both of these salts cause significant decrease of GPx levels, resulting in high oxidative stress. In MgSO_4_ treated mice the GPx level reduced substantially, making it more lethal than K_2_Cr_2_O_7_.

**Fig.1:**
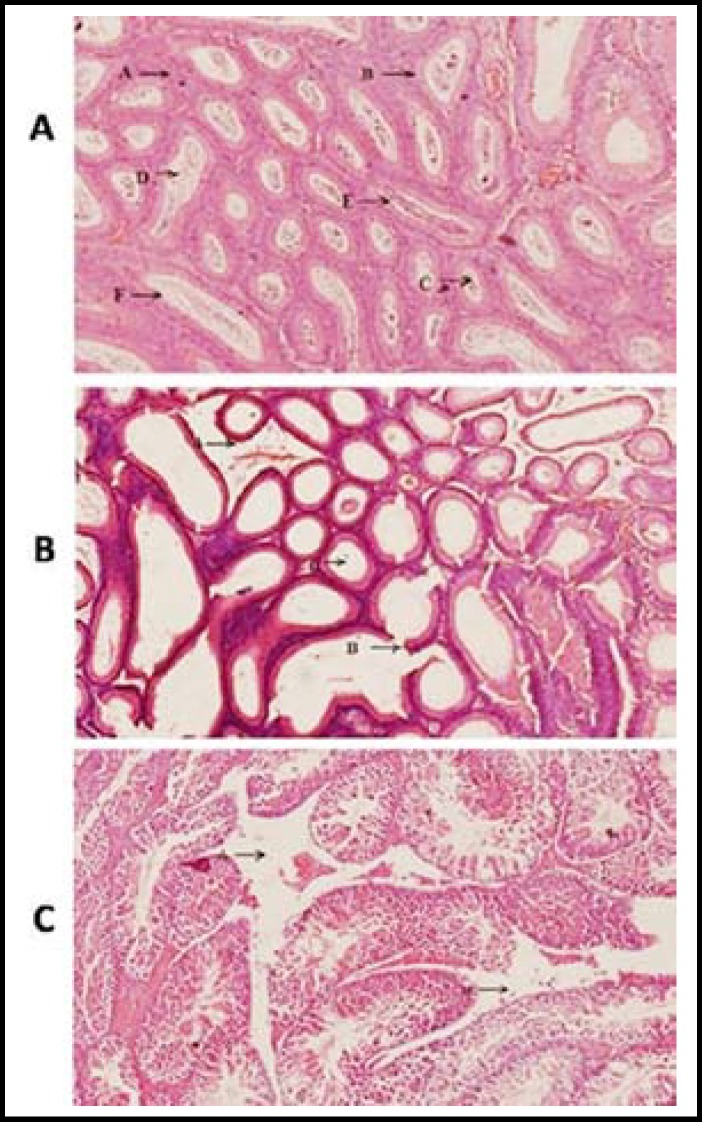
**a)** Cross section of testicle of mice of control group at 30^th^ day of experiment. A. Leydig Cells, B. Germinal Layer, C. Spermatids, D. Spermatocytes, E. Spermatogonia, F. Sertoli cell.

**Fig.2:**
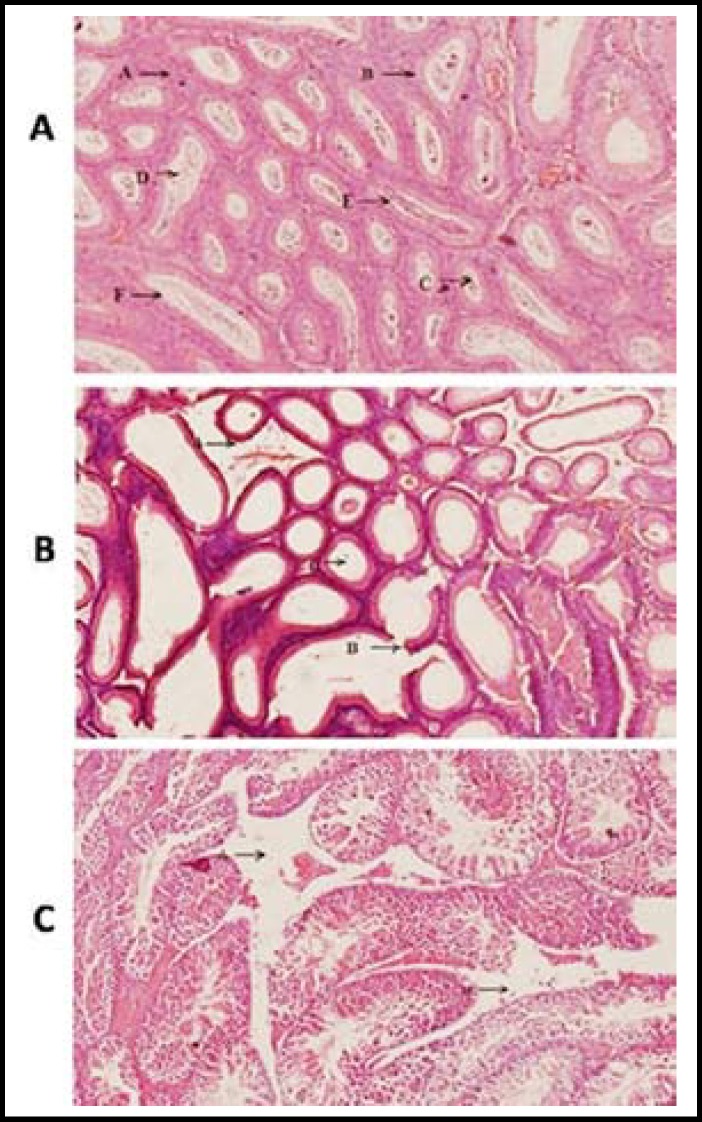
**a)** Cross section of testicle of mice of control group at 60^th^ day of experiment. A. Leydig Cells, B. Germinal Layer, C. Spermatids, D. Spermatocytes, E. Spermatogonia, F. Sertoli cell.

***Morphological analysis of Testicular tissue: ***We also studied the morphology of the cross sectional structure of testicles of normal follicles with intact germinal layer and leydig cell and the results showed complete destruction of leydig cells, partially damaged germinal layer and destruction of sperms leading to empty spaces in the lumen of follicle of testicles in which 30% destruction of follicle was observed in the group at 30^th^ day after the administration of K_2_Cr_2_O_7 _and 35% destruction of follicle was observed in the group at 30^th^ day after administration the MgSO_4_ (Fig. 1a, b, c). Furthermore, 55% destruction of follicle was observed in this cross section of group B at 60^th^ day after administration the K_2_Cr_2_O and 64% destruction of follicle was observed in this group at 60^th^ day after administration the MgSO_4_ (Fig. 2a, b, c).

## DISCUSSION

Hexavalent chromium is very toxic and readily enters the cells and induces toxicity provoke lipid peroxidation, DNA damage, cytotoxicity, mutagenesis and carcinogenesis. MgSO_4_ effects on the lipid peroxidation and significantly increases MDA.^3^^,^^6^ The results of lipid peroxidation, MDA, SOD, CAT and GPx showed significant difference in MDA, SOD, CAT and GPx levels in K_2_Cr_2_O_7_ and MgSO_4_ administrated group after 30^th^ and 60^th^ days treatment. The outcomes were in line with the Goulart *et al*.^17^. However, further research is needed to estimate the effect of MgSO_4_ on lipid peroxidation. In K_2_Cr_2_O_7_ and MgSO_4 _administrated group ROS were produced and these reactive molecules are able to remove hydrogen from the lipid membrane and imitating a series of reaction leading to the membrane destruction.

**Table-Ia:** MDA levels in different groups at different days

***Days***	***Groups***	***Means ± SD (*** *μmol/ml* ***)***	***(P-Value)***
1^st^ day	A B C	24.64+.34 24.16+.30 24.48+0.56	0.426
30^th^ day	A B C	24.49+0.31 26.80+0.76 26.57+0.20	0.004*
60^th^ day	A B C	24.50+0.62 27.69+0.25 26.78+0.21	0.001*

* Significant (p < 0.05)

**Table-Ib:** SOD levels in different groups at different days

***Days***	***Groups***	***Means ± SD (*** *μg/ml* ***)***	***(P-Value)***
1^st^ day	A B C	28.77+1.18 28.70+0.65 28.27+0.64	0.329
30^th^ day	A B C	28.35+1.13 26.16+0.43 19.43+2.04	0.004*
60^th^ day	A B C	28.51+0.48 22.82+0.91 22.44+5.08	0.001*

* Significant (p < 0.05)

**Table-IIa:** Catalase levels in different groups at different days

***Days***	***Groups***	***Means ± SD (*** *μmol/mol of protein* ***)***	***(P-Value)***
1^st^ day	A B C	191.47+0.62 190.41+0.69 191.29+0.44	0.147
30^th^ day	A B C	190.78+0.98 183.63+6.70 173.30+2.79	0.357
60^th^ day	A B C	190.52+0.94 186.52+1.32 179.53+4.08	0.005*

* Significant (p < 0.05)

**Table-IIb:** Glutathione peroxidase levels in different groups at different days

***Days***	***Groups***	***Means ± SD*** ***(µmol/g protein)***	***(P-Value)***
1^st^ day	A B C	33.5+0.62 32.7+0.69 34.9+0.44	0.321
30^th^ day	A B C	31+0.98 19.9+6.70 09.7+2.79	0.005*
60^th^ day	A B C	31.4+0.94 15.3+1.32 07.7+4.08	0.001*

* Significant (p < 0.05)

**b) **Cross section of testicle of mice of group B at 30^th^ day. A. Destruction of Leydig cells, B. Partially damaged germinal layer, C. Empty central lumen of follicle of testis without sperms.

**c) **Cross section of testicle of mice of group C at 30^th^ day. A. Destruction of Leydig cells B. partially damaged germinal layer.

**b) **Cross section of testicle of mice of group B at 60^th^ day. A. Destruction of Leydig cells and D. destruction of complete follicles.

**c) **Cross section of testicle of mice of group C at 60^th^ day. A. Destruction of Leydig cells, B. Empty central lumen of follicle of testis without sperms, C. partially damaged germinal layer D. destruction of complete follicle.

Salts of heavy metals when ingested in high dose produced oxygen reactive species which may lead to adverse clinical outcomes such as DNA damage, destruction of testicular cells and germ line of testis at initial levels. Findings of the present study represent similar results by investigating the destruction of germ cells, spermatocytes, spermatogonia, sertoli cells, sperms and ultimate’s whole follicles after administration of the both the salts for two months. However, heavy destruction was observed in the MgSO_4_ administrated group (being 35% and 66% at 30^th^ and 60^th^ day respectively) as compared to K_2_Cr_2_O_7_ administrated group where 30% destruction of follicles were observed at 30^th^ day and 56% destruction of follicle at 60^th^ day of experiment as previously explained by Acharya *et al*.^9^

The destructive effects of both the salts on testicles of male mice could be due to generation of ROS in exposure to the K_2_Cr_2_O_7_ and MgSO_4_. Oxygen reactive species generated are active in causing DNA damage. The DNA damage in soft tissue of follicles leads to excessive destruction of follicles, germ line leading to severe reproductive abnormality resulting in production of sterile individuals.^18^ The significant changes in lipid peroxidation and testicular histology suggested that K_2_Cr_2_O_7_ and MgSO_4_ exposure increase the level of lipid peroxidation leading towards generation of ROS and severe destructive consequences of testicular cells in male mice.

## CONCLUSION

We conclude that K_2_Cr_2_O_7_ and MgSO_4_ have a deleterious effect on the histology of the testis of male mice. The effects of MgSO_4 _found to be more lethal and destructive as compared to K_2_Cr_2_O_7_. Thus, we suggest further detailed studies in human to corroborate these findings and assuming that K_2_Cr_2_O_7_ and MgSO_4_ at normal dose could be a potential male anti fertility agent. The self medication involving K_2_Cr_2_O_7_ and MgSO_4_ should also be discouraged.

## Authors Contributions:

*AM, KZ and MR:* Designed the study. *UH, AM, and MA:* Collected the data and performed the experiments. *MHQ, MIN, MR and AM:* Analyzed the data critically, performed statistical analysis and wrote the manuscript.
